# Quality of Life in Older Adults: Evidence from Mexico and Ecuador

**DOI:** 10.3390/geriatrics6030092

**Published:** 2021-09-16

**Authors:** Paola Ochoa Pacheco, Rafael Castro Pérez, David Coello-Montecel, Nancy Pamela Castro Zazueta

**Affiliations:** 1 ESPAE Graduate School of Management, Escuela Superior Politécnica del Litoral (ESPOL), Campus Las Peñas Malecón No. 100 y Loja, Guayaquil 090306, Ecuador; 2 Facultad de Psicología, Universidad Autónoma de Sinaloa, Ciudad Universitaria Culiacán, Culiacán 80013, Mexico; rafaelcastroperez@uas.edu.mx (R.C.P.); p.castro@uas.edu.mx (N.P.C.Z.)

**Keywords:** quality of life, older adults, Mexico, Ecuador

## Abstract

Older adults are a growing population group in Latin America, hence the importance of deepening studies, proposals, and policies to guarantee their well-being. This article analyzes the perception of quality of life in older adults from Mexico and Ecuador and its association with several socioeconomic variables. The study design was cross-sectional. The sample comprised 450 older adults, 238 from Mexico and 212 from Ecuador. The WHOQOL-OLD Quality of Life Questionnaire and a set of sociodemographic variables were used. The results showed a higher perception of quality of life in the Mexican sample regarding most of the dimensions, except for sensory skills and social participation. An association was also found between sensory skills and sports practice, as well as between social participation and education level. The study achieves a binational approach to the reality of older adults in Latin America and confirms that there are differences in each sample that are due to the particularities of each reality. This research contributes to deepening the reality of the elderly, especially in Ecuador, where the quality-of-life studies in all age segments must be strengthened.

## 1. Introduction

Older adults represent a little more than 9.3% of the world population, and by 2050 this proportion will increase to 16% [1]. According to the Inter-American Development Bank [2], Latin America and the Caribbean will be the region with the highest growth rate of the elderly population, representing approximately 25% of the total population by 2050. By that year, according to regional projections, this population will have grown faster in countries such as Brazil, Colombia, Mexico, and Ecuador. The growth of the elderly population worldwide has exposed outstanding deficiencies, opportunities, and challenges that have arisen due to the social and economic changes experienced in recent years. Among the challenges that the Latin-American countries will have to face in the coming years are the need to expand the access to health services, the sustainability of the social security systems, the coexistence of high levels of poverty with reduced capacities to undertake new projects, and inequity [3,4].

In addition to the rapid population growth, another factor determining the challenges of the Latin-American countries to guarantee well-being and quality of life in their elderly population is the high and growing prevalence of chronic diseases. Regarding this aspect, factors such as sedentary lifestyle and poor eating habits have been identified as predictors of the increase in the prevalence of diabetes in Mexico or musculoskeletal diseases in Ecuador [2]. On this line, regional statistics place Mexico and Ecuador among the countries with the highest proportion of dependent older adults, with difficulties in carrying out some basic daily life activities. Furthermore, the economic environment in countries such as Ecuador and Mexico has not provided conditions for improving the quality of life of the elderly in both countries, with high rates of moderate poverty in the elderly population and limited access to a retirement pension [2] being some of the indicators of their vulnerability.

In Mexico, the elderly represent approximately 9% of the total population according to the National Population Council, and by 2050 one out of every five inhabitants will be part of this population group. In the Aztec country, any person aged 60 or more is considered an older adult according to the current legislation. One-third of the elderly are economically active; however, approximately 40% of older adults live in poverty [5]. Of the elderly population in Mexico, 23.5% receives a contributory pension, and 32.9% receives a non-contributory pension [2].

The Ecuadorian regulation establishes that any person over 65 is considered an older adult in Ecuador. This group represents a little more than 9% of the Ecuadorian population. According to data from the National Employment Survey (ENEMDU by its acronym in Spanish) [6] published in December 2020, 63% of Ecuadorian older adults are not economically active, while those who are economically active primarily have unsuitable jobs (23.6%), unpaid jobs (4.9%), or are underemployed (3.3%). At the national level, 45.7% of the elderly are affiliated or covered by some type of insurance (public or private), of which 71.5% have access to a retirement pension. Among Ecuadorian social security pensioners, 65% earn a monthly income below the minimum wage (400 dollars in 2020). In addition, 18% of Ecuadorian older adults live in poverty or extreme poverty conditions.

Considering the Latin-American context and focusing on the reality of the elderly in Mexico and Ecuador—in which high prevalence of chronic diseases, a high number of older adults in dependency conditions, and low access to a retirement pensions are issues of concern—this article aims to compare the perception of quality of life in older adults from both countries, as well as their association with a set of sociodemographic variables.

Quality of life is associated with several dimensions of well-being. Its measurement includes aspects such as the physical, mental, and social state of a person or a group of people [7]. In the context of the elderly, quality of life refers to the ability to participate in society and enjoy and feel pleasure from it [8], regardless of the presence or absence of disease [9]. It is also defined as the perception of the elderly about the enjoyment of health, sufficient food, decent housing, equality, dignity, and security. Beyond basic needs, authors such as Sadana et al. [3] propose to discuss facilities and services that contribute to raising the standard of living of older people such as infrastructures, opportunities for walking and exercise, social inclusion, reducing discriminatory practices, diminished exposure to risk factors associated with communicable and non-communicable diseases, inclusion in insurance schemes, and prevention of elder abuse.

This research started from the definition of ‘quality of life’ proposed by the World Health Organization in 1995, which is understood as the individual’s belief about his position in life within his cultural context and values concerning his goals, expectations, standards, and concerns. According to the WHOQOL-OLD Quality of Life Questionnaire of the World Health Organization, quality of life comprises the following dimensions: sensory abilities; autonomy; past, present, and future; social interaction; death and dying; and intimacy [10]. The ‘sensory abilities’ dimension refers to the functioning of the senses and the impact of the loss of sensory abilities on quality of life. The ‘autonomy’ dimension measures the sense of independence, the perception or belief of feeling free to live autonomously and make own decisions. For its part, the ‘past, present, and future’ dimension describes the satisfaction of the elderly concerning the achievements made during their life. The ‘social interaction’ dimension, in turn, refers to participation in daily life activities, especially those that are developed within the community. The ‘death and dying’ dimension reflects the concerns and fears of the elderly about death. Finally, the ‘intimacy’ dimension measures the ability of the elderly to maintain personal relationships with other individuals, be they members of their family nucleus, romantic partner, friends, or other people.

The existing literature has explored the association of quality of life with several factors such as health conditions [11,12,13,14], physical exercise [15,16,17,18,19,20,21], social support and social relationships [22,23,24], income [25,26], educational level [27,28,29], leisure activities [30,31,32], and productive activities after retirement [33], among others. Moreover, there are numerous elements at the individual level—such as self-esteem and optimism [34], anxiety and depression [35], or pain and dignity [36]—that must be taken into account due to their impact on quality of life in older adults.

Important meta-analyses on the quality of life in older adults [37,38], and there are numerous studies with different emphasis in Latin America regarding the quality of life of the elderly [29,39,40,41,42]. Among them, Vitorino et al. [40] found that a higher perception of quality of life is associated with a higher level of schooling and the practice of physical and leisure activities. González-Celis & Padilla [39], for their part, found that older adults with some disease reported lower perception of quality of life.

Several articles have explored the factors associated with quality of life in Mexican older adults [43,44,45,46], although some focused on health-related quality of life [47,48]. Based on the evidence of a sample of older adults from the State of Sonora, Acosta et al. [43] determined that social, marital, and family loneliness negatively affect quality of life. Similar results were found in the study conducted by González-Celi & Lima [44] on older adults from a public health clinic in the State of Mexico. For their part, Gutiérrez-Vega et al. [46] analyzed the relationship between the marital status and the quality of life of older adults from the city of Juárez, concluding that there is a greater perception of quality of life in married older adults, compared to single or divorced, in the psychological (e.g., self-esteem, positive and negative feelings) and social relationships (e.g., social support, personal relationships) dimensions. Soria-Romero & Montoya-Arce [45] found that access to health care services, better housing conditions, and happiness are associated with a higher sense of quality of life.

In Ecuador, the study on the quality of life in older adults has begun, but in an incipient way [49,50,51,52,53,54,55,56,57]. Bustamante et al. [49] measured quality of life as a one-dimensional construct and found that a greater sense of quality of life is associated with job type and educational level. For their part, Gordillo Altamirano et al. [50] determined that physical functionality is associated with a greater perception of quality of life in terms of physical health, while factors such as anxiety and depression negatively affect the quality of life of older adults, specifically their mental health. Guevara et al. [56] concluded that the prevalence of rheumatic diseases influences a lower quality of life in indigenous older adults. In this sense, the present study differs from others carried out in the elderly population of Ecuador since it considers the multidimensionality of the quality of life and incorporates numerous sociodemographic factors that have not been analyzed in this population (e.g., sports practice, the performance of medical examinations).

This study provides evidence on the scarce literature on the quality of life of older adults in Ecuador and Mexico and useful inputs for formulating public policies and effective interventions. Additionally, within health and social sciences research in Latin America it makes a population group visible that, in the medium term, due to a longer life span, will represent new challenges for health care and sustainability. We consider the study innovative given the scarcity of updated official statistics regarding older adults’ quality of life, especially in one of the study countries, Ecuador. In the case of Ecuador, it is challenging to obtain specialized, standardized, and comparable data or indicators that reflect the current situation of this population group. In addition, this study provides a baseline for a better understanding of the living conditions of the elderly in developing countries such as Ecuador. In this country, except for the 2009 Elderly Health and Well-being National Survey carried out by the National Institute of Statistics (INEC by its acronym in Spanish), the empirical evidence on the quality of life and psychosocial well-being in this population group is still incipient. Finally, our study contributes to the existing literature since, to our best knowledge, studies on the quality of life of older adults in Latin-American countries with a comparative approach are limited.

## 2. Materials and Methods

### 2.1. Participants

The study design was cross-sectional. The sample was incidental and comprised 450 older adults, of which 238 (52.89%) were from Mexico and 212 (47.11%) from Ecuador (Table 1). The sample from Mexico was composed mainly of men (81.09%) between 60 and 69 years old (82.77%). Most of the Mexican participants indicated that they had completed graduate studies (61.76%), are married (84.03%), have an unpaid job (70.59%), are not diagnosed with any chronic disease (61.76%), medical examinations are carried out up to twice a year (70.81%), and practice sports regularly (65.97%). For its part, the Ecuadorian sample consisted mainly of women (85.38%) between 70 and 79 years old (62.69%). The majority of the respondents reported having completed high school (46.23%) and undergraduate (33.49%) studies and have an unpaid job (87.56%). 65.09% of the participants in this study are married, diagnosed with a chronic disease (65.57%), undergo medical examinations more than twice a year (41.51%), and play sports regularly (68.40%).

### 2.2. Measures

The Spanish version [58] of the WHOQOL-OLD Quality of Life Questionnaire from the World Health Organization [10] was used to measure the quality of life. This questionnaire has been validated in different countries of Europe (e.g., Portugal [59], Germany [60], France [61]), Asia (e.g., Turkey [62], Iran [63], China [64]), Africa (e.g., Ghana [65]), and Latin America (e.g., Brazil [66,67], Chile [68], Mexico [41], Peru [69]).

The WHOQOL-OLD questionnaire comprises 24 items grouped into six dimensions: sensory abilities; autonomy; past, present, and future; social interaction; intimacy; and death and dying. Items were rated using a five-point Likert scale, from 1 to 5; higher scores represented a higher perception of quality of life. Sample items include: “To what extent does the loss of, for example, hearing, vision, taste, smell or touch affect your ability to participate in activities?” (sensory abilities); “How much freedom do you have to make your own decisions?” (autonomy); “To what extent are you satisfied with your opportunities to continue achieving in life?” (past, present, and future); “How satisfied are you with your opportunity to participate in community activities?” (social interaction); “How concerned are you about the way in which you will die?” (death and dying); “To what extent do you experience love in your life?” (intimacy). The mean of the items that constitute each dimension, as well as the sum of the items scores, are reported in the results section.

To evaluate the construct validity of the WHOQOL-OLD questionnaire, as well as to validate its factorial structure, a series of confirmatory factor analyses were performed using both Mexican and Ecuadorian samples. The goodness-of-fit of each of the specifications was analyzed with the comparative fit index (CFI), the goodness-of-fit index (GFI), the Tucker-Lewis index (TLI), the root mean square error of approximation (RMSEA), and the standardized root mean square residual (SRMR), instead of the chi-square to degrees of freedom ratio (χ^2^/gl) since it is sensitive to sample size. Internal consistency was assessed using the McDonald’s omega (ω) composite reliability coefficient rather than the Cronbach’s alpha (α) coefficient in order not to underestimate reliability when there is considerable variation in factor loadings [70,71,72].

All of the model fit indices were adequate for both Mexican (CFI = 0.928; GFI = 0.883; TLI = 0.910; RMSEA = 0.074 (90% CI: 0.074–0.063); SRMR = 0.073) and Ecuadorian samples (CFI = 0.937; GFI = 0.880; TLI = 0.923; RMSEA = 0.064 (90% CI: 0.044–0.070); SRMR = 0.068). All factor loadings were statistically significant (*p* < 0.001). The reliability estimates of the WHOQOL-OLD dimensions for the Mexican sample ranged between 0.714–0.894, while for the Ecuadorian sample ranged between 0.654–0.940, indicating a satisfactory internal consistency [73].

### 2.3. Data Collection Procedure

Data collection occurred between February and June 2017 in Sinaloa (Mexico) and Cuenca and Guayaquil (Ecuador). Before delivering the questionnaire, the study’s objectives were explained to the older adults who agreed to participate. Data collection was approved by the educational and geriatric centers that collaborated with the investigation.

### 2.4. Statistical Analyses

Scoring for each of the WHOQOL-OLD dimensions was computed considering the average of the items (1–5 scale range), the raw sum of the items (4–20 scale range), and a transformed score (0–100 scale range) [10]. As indicated in Section 2.2, the construct validity of the WHOQOL-OLD questionnaire was evaluated using confirmatory factor analysis, and reliability was assessed using the McDonald’s omega composite reliability coefficient. To determine the existence of statistically significant differences in the WHOQOL-OLD dimensions scores between Mexican and Ecuadorian older adults, mean comparison tests were performed (*t*-test) using the mean score of the items that comprise each dimension. Mean comparison tests were carried out to determine an association between the WHOQOL-OLD dimensions and the sociodemographic variables that were included in the study. A *t*-test was performed to compare the scores among older adults grouped by gender, age (only for the Mexican sample), educational level (only for the Mexican sample), marital status, work after retirement, presence of chronic disease, and sports practice. On the other hand, an ANOVA analysis was carried out to compare the scores of older adults according to age (only for the Ecuador sample), educational level (only for the Ecuador sample), and frequency of medical examinations. Statistical analyses were executed using AMOS Version 24 and IBM SPSS Statistics Version 26.

## 3. Results

**Figure 1:**
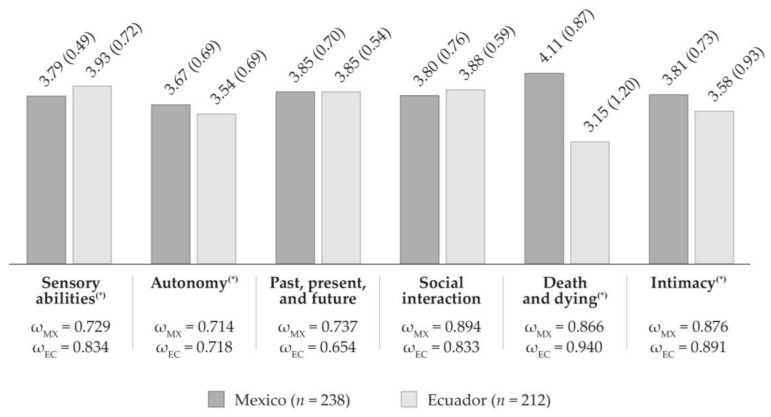
Descriptive statistics and composite reliability estimates of the WHOQOL-OLD dimensions among countries. Notes—The vertical bar graph reports the mean scores of the WHOQOL-OLD dimensions (1–5 scale range). Standard deviations are reported in parenthesis. The raw sum scores for the Mexican sample (4–20 scale range) were: 15.16 (sensory abilities), 14.69 (autonomy), 15.40 (past, present, and future), 15.21 (social interaction), 16.42 (death and dying), and 15.23 (intimacy). The raw sum scores for the Ecuadorian sample (4–20 scale range) were: 15.71 (sensory abilities), 14.17 (autonomy), 15.41 (past, present, and future), 15.53 (social interaction), 12.61 (death and dying), and 14.31 (intimacy). In Mexico, the transformed scores (0–100 scale range) were: 69.72 (sensory abilities), 66.83 (autonomy); 71.24 (past, present and future); 70.09 (social interaction), 77.63 (death and dying), and 70.19 (intimacy); while, in Ecuador they were: 73.17 (sensory abilities), 63.59 (autonomy), 71.31 (past, present and future), 72.08 (social interaction), 53.80 (death and dying), and 64.45 (intimacy). All scores and standard deviations were calculated using all indicators of the corresponding dimension. ω: McDonald’s omega composite reliability estimates. (*): Between both countries, there were found statistically significant differences at *p* > 0.05.

Figure 1 shows the mean scores and the composite reliability estimates for each of the WHOQOL-OLD dimensions for Mexican and Ecuadorian samples. It denotes the statistically significant differences found between both countries. Mexican older adults reported a higher quality of life scores in autonomy, death and dying, and intimacy dimensions, while Ecuadorians indicated a higher perception of quality of life concerning their sensory abilities and social interaction. Statistically significant differences were found between both countries in sensory abilities (*t* = 2.39; *p* < 0.05), autonomy (*t* = 1.99; *p* < 0.05), death and dying (*t* = 9.70; *p* < 0.01), and intimacy (*t* = 2.93; *p* < 0.01) dimensions. The reliability estimates for the Mexican sample averaged 0.803 (ranging from 0.714 to 0.894) and 0.812 for the Ecuadorian sample (ranging from 0.654 to 0.940).

Table 2 and Table 3 show the mean scores of the WHOQOL-OLD dimensions for older adults of both countries by sociodemographic variables. The corresponding *p*-value of the mean comparison tests that were carried out is also reported. The statistically significant differences found between Mexican and Ecuadorian older adults are discussed below.

In the Mexican sample, statistically significant differences were found in the WHOQOL-OLD dimensions when comparing by age, frequency of medical examinations, and sports practice. Although when comparing the scores by gender, no statistically significant differences were found, women scored higher than men in the autonomy and intimacy dimensions. In contrast, men scored higher than women in sensory abilities, social interaction, and death and dying dimensions. Regarding the comparison by age, significant differences were determined in both autonomy and death and dying dimensions. Older adults between 60 and 79 years reported a greater degree of autonomy as well as a higher concern about death than the other Mexican older adults.

Also, in Mexico, the scores in autonomy; past, present, and future activities; social interaction, death and dying, and intimacy dimensions were higher in those older adults who attended medical examinations more frequently. When comparing the dimensions scores by the frequency of medical examinations, significant differences were determined in social interaction and autonomy dimensions.

Older adults who regularly practice sports reported a greater perception of autonomy, greater satisfaction with the achievements achieved during their life, greater social interaction, and higher scores in the intimacy dimension than those who do not practice sports. However, significant differences were found only in the intimacy dimension.

In the case of Ecuador, significant differences were determined when comparing by gender, age, educational level, civil status, work activity after retirement, frequency of medical examinations, and sports practice. Women reported greater sensory abilities than men, a higher degree of satisfaction about past, present, and future, and higher scores in social interaction, death and dying, and intimacy dimensions. Male Ecuadorian participants self-evaluated with a greater degree of autonomy. Significant differences were found between men and women in the death and dying dimension.

Statistically significant differences were also found in autonomy, sensory abilities, and death and dying dimensions when comparing by age. Older adults between 60 and 79 years old reported higher scores in all dimensions of quality of life than older adults who were 80 years or older.

Ecuadorian older adults who completed graduate studies reported themselves as more autonomous, with greater satisfaction regarding their past, present, and future, and greater social interaction. Significant differences were found in autonomy; social interaction; and past, present, and future dimensions when comparing WHOQOL-OLD dimensions by educational level.

Moreover, older adults who have a paid job indicated higher scores in autonomy; past, present, and future; and social interaction dimensions. Those who carry out unpaid activities expressed greater concern about death. Significant differences were found only in the death and dying dimension.

Those older adults who attend medical examinations less frequently reported higher scores in the sensory abilities dimension and also reported higher perceptions of autonomy; satisfaction with present, past, and future; and social interaction. When comparing by the frequency of medical examinations, statistically significant differences were found only in the sensory abilities dimension.

Finally, it was observed that older adults who play sports presented higher scores in all dimensions of quality of life; however, there were statistically significant differences only in the sensory abilities dimension.

## 4. Discussion

The objective of this article was to analyze the perception of quality of life in older adults from Mexico and Ecuador and its association with several socioeconomic variables. In this sense, two main findings will be discussed in this section. First, there were significant differences in some of the dimensions of quality of life between Mexican and Ecuadorian older adults. Second, in Ecuador, there was an association between greater sensory abilities and sports practice, as well as between a greater sense of social interaction and a higher level of education.

Regarding the first finding, the results showed differences between older adults from both countries in terms of quality of life. The higher scores of the Mexican participants in the autonomy dimension may be related to their characteristics since they were younger and reported better health status than the Ecuadorian participants. However, generalizations cannot be made since the Ecuadorian older adults scored slightly higher in another fundamental dimension of quality of life, such as sensory abilities. The results regarding the quality of life of the older adults that participated in this study are consistent with regional data presented by institutions such as the Inter-American Development Bank [2]. Those statistics provide evidence of a high prevalence of chronic diseases (e.g., diabetes, coronary heart disease, and musculoskeletal diseases) and a significant number of older adults in a dependency condition in countries such as Mexico and Ecuador, which could explain the scores reported in some dimensions, such as autonomy. Some other biopsychosocial factors should be incorporated in further research to characterize in a better way the lifestyle of the elderly in both countries and deepen explanations about the determinants of these differences beyond the statistical analysis.

Our findings on the dimensions of quality of life in older adults of both countries were consistent with previous studies carried out in other Latin-American countries such as Brazil [74], Chile [68], Peru [42,75], and Mexico itself [41]. Specifically, the levels of quality of life of the Mexican older adults reported in our study are similar to those shown by González-Celis and Gómez-Benito [41]. On the other hand, the perception of quality of life indicated by the Ecuadorian and Mexican older adults participating in our study was higher than the results obtained by Urzúa and Navarrete in Chile [68] and Queirolo et al. [42] and Hernandez-Huayta et al. [75] in Peru.

The second finding raises the characteristics within the sample of each country. Some aspects found in the Ecuadorian sample should be highlighted, such as that those older adults who attend medical examinations less frequently reported higher scores in the sensory abilities dimension. Higher sensory abilities were also found in those who practice sports, following the existing literature that establishes that physical activity promotes health, improves living conditions, and prevents diseases such as muscle atrophy, sarcopenia, osteoporosis, type 2 diabetes, or coronary diseases [16,17,18,19,20,76]. According to the extensive literature that has addressed this issue [37,38], physical exercise prevents chronic diseases such as diabetes or cardiovascular diseases, which would affect older adults’ sensory abilities.

Our findings also supported an association between educational level and social participation, given that those older adults with undergraduate or graduate studies reported a greater sense of social participation. In this sense, the findings of our study are consistent with previous research [28,40,77], which suggests that a higher level of education allows the elderly to have more options, visualize opportunities, or even undertake productive activities after retirement or even re-enter the labor market.

According to our results, a greater sense of social interaction was evidenced in those older adults who have a paid job after retirement. In this sense, there are different opinions regarding the effect that retirement has on quality of life. On the one hand, some authors suggest that it is often expected that, when they stop working, the social coexistence networks established around work begin to lose strength and progressively decrease; thus, social exclusion as a consequence of disengagement work contributes to a gradual loss of well-being. On the other hand, continuing a work activity after retirement promotes the maintenance or construction of social relationships, positive emotions, and successful aging. Our results provide evidence for this second position and are aligned with the existing literature [23] that provides evidence on the importance of social relationships for improving older adults’ quality of life.

The study has some limitations because of the use of an incidental sample and self-report measures. However, it also has several strengths. First, it provides a baseline for a better understanding of the living conditions of the elderly in developing countries such as Ecuador and Mexico. Second, it offers empirical results about older adults’ quality of life in two Latin-American countries. Third, the study’s findings are useful inputs for social and health intervention proposals, more effective public policies, and a discussion about improving older adults’ quality of life to build a more sustainable society. The study is the first stage for the cross-cultural validation process of the WHOQOL-OLD Quality of Life questionnaire. As a psychosocial study, the objective was not to generalize results, but to obtain evidence that allows deepening the knowledge of older adults’ psychological and social characteristics and their situation of vulnerability in Latin-American countries.

## Figures and Tables

**Table 1:** Sociodemographic characteristics.

Variable	Mexico–*n* (%)	Ecuador–*n* (%)	Overall Sample–*n* (%)
Gender			
Male	193 (81.09)	31 (14.62)	224 (49.78)
Female	45 (18.91)	181 (85.38)	226 (50.22)
Age	64.57 ± 4.51	73.14 ± 5.73	68.61 ± 6.67
Between 60–69 years	197 (82.77)	49 (23.11)	246 (54.67)
Between 70–79 years	41 (17.23)	132 (62.26)	173 (38.44)
More than 80 years	-	31 (14.62)	31 (6.89)
Education			
Elementary school	-	22 (10.38)	22 (4.89)
High school	-	98 (46.23)	98 (21.78)
Undergraduate	91 (38.24)	71 (33.49)	162 (36.00)
Graduate	147 (61.76)	21 (9.91)	168 (37.33)
Civil status			
Married	200 (84.03)	138 (65.09)	274 (60.89)
Single (a)	38 (15.97)	74 (34.91)	176 (39.11)
Work after retirement			
Paid job	70 (29.41)	25 (12.44)	95 (21.64)
Unpaid job	168 (70.59)	176 (87.56)	344 (78.36)
Chronic disease			
Yes	91 (38.24)	139 (65.57)	230 (51.11)
No	147 (61.76)	73 (34.43)	220 (48.89)
Frequency of medical examinations			
More than twice a year	61 (29.19)	88 (41.51)	149 (35.39)
Once or twice a year	71 (33.97)	53 (25.00)	124 (29.45)
Less than once a year	77 (36.84)	71 (33.49)	148 (35.15)
Sport practice			
Yes	157 (65.97)	145 (68.40)	302 (67.11)
No	81 (34.03)	67 (31.60)	148 (32.89)

(a): Includes single, divorced, and widowed.

**Table 2:** Mean comparison of the WHOQOL-OLD dimensions among countries by sociodemographic characteristics. First part.

Variable	Sensory Abilities		Autonomy		Past, Present, and Future	
	MX	EC	MX	EC	MX	EC
Gender						
Male	4.35 (0.67)	3.98 (0.87)	3.65 (0.76)	3.76 (0.63)	3.99 (0.77)	3.84 (0.56)
Female	4.27 (0.79)	4.18 (0.86)	3.80 (0.65)	3.55 (0.76)	3.99 (0.71)	3.85 (0.53)
*p*-value	0.496	0.219	0.224	0.140	0.997	0.876
Age						
Between 60–69 years	4.32 (0.69)	4.05 (0.90)	3.63 (0.73)	3.79 (0.77) ^1^	3.97 (0.76)	3.94 (0.53)
Between 70–79 years	4.41 (0.71)	4.27 (0.81) ^1^	3.89 (0.76)	3.56 (0.78)	4.11 (0.77)	3.82 (0.56)
More than 80 years	-	3.83 (0.91) ^1^	-	3.35 (0.47) ^1^	-	3.86 (0.41)
*p*-value	0.485	<0.05	<0.05	<0.05	0.297	0.363
Education						
Elementary school	-	3.82 (0.82)	-	3.14 (0.78) ^1,2,3^	-	3.66 (0.55) ^1^
High school	-	4.28 (0.80)	-	3.60 (0.75) ^1^	-	3.88 (0.56)
Undergraduate	4.28 (0.72)	4.11 (0.97)	3.59 (0.76)	3.59 (0.70) ^2^	3.97 (0.78)	3.80 (0.49) ^2^
Graduate	4.37 (0.68)	4.10 (0.72)	3.73 (0.72)	3.92 (0.65) ^3^	4.01 (0.75)	4.11 (0.50) ^1,2^
*p*-value	0.301	0.131	0.159	<0.01	0.679	<0.05
Civil status						
Married	4.32 (0.70)	4.27 (0.75)	3.66 (0.74)	3.59 (0.66)	3.97 (0.76)	3.85 (0.53)
Single(a)	4.43 (0.68)	4.09 (0.91)	3.78 (0.73)	3.57 (0.79)	4.11 (0.74)	3.86 (0.54)
*p*-value	0.372	0.150	0.357	0.838	0.321	0.927
Work after retirement						
Paid job	4.29 (0.65)	4.41 (0.60)	3.75 (0.83)	3.81 (0.93)	4.13 (0.69)	3.99 (0.57)
Unpaid job	4.36 (0.71)	4.12 (0.87)	3.65 (0.70)	3.55 (0.72)	3.93 (0.78)	3.84 (0.53)
*p*-value	0.500	0.101	0.358	0.097	0.066	0.194
Chronic disease						
Yes	4.26 (0.74)	4.11 (0.88)	3.70 (0.69)	3.60 (0.74)	3.94 (0.74)	3.82 (0.54)
No	4.38 (0.66)	4.25 (0.81)	3.67 (0.77)	3.53 (0.76)	4.03 (0.77)	3.91 (0.53)
*p*-value	0.197	0.257	0.785	0.518	0.378	0.225
Frequency of medical examinations						
More than twice a year	4.28 (0.74)	3.96 (0.93) ^1^	3.70 (0.74)	3.53 (0.69)	4.03 (0.64)	3.77 (0.54)
Once or twice a year	4.33 (0.73)	4.23 (0.79)	3.80 (0.76) ^1^	3.52 (0.81)	4.12 (0.76)	3.91 (0.56)
Less than once a year	4.35 (0.64)	4.33 (0.77) ^1^	3.48 (0.73) ^1^	3.69 (0.77)	3.84 (0.85)	3.91 (0.52)
*p*-value	0.847	<0.05	<0.05	0.346	0.075	0.189
Sport practice						
Yes	4.37 (0.71)	4.24 (0.85)	3.71 (0.77)	3.60 (0.77)	4.06 (0.72)	3.89 (0.53)
No	4.28 (0.66)	3.98 (0.86)	3.63 (0.68)	3.53 (0.70)	3.86 (0.81)	3.78 (0.55)
*p*-value	0.393	<0.05	0.421	0.486	0.052	0.180

MX: Mexico; EC: Ecuador. (a): Includes single, divorced, and widowed older adults. Standard deviations are shown in parentheses. A *t*-test was used to compare the dimensions of quality of life among older adults grouped by gender, age (only for the Mexican sample), educational level (only for the Mexican sample), marital status, work after retirement, presence of chronic disease, and sports practice. An ANOVA test was performed to compare the scores of older adults according to age (only for the Ecuador sample), educational level (only for the Ecuador sample), and frequency of medical examinations. ^1^, ^2^, ^3^: Statistically significant differences were found between these pairs at a *p* <0.05 level.

**Table 3:** Mean comparison of the WHOQOL-OLD dimensions among countries by sociodemographic characteristics. Second Part.

Variable	Social Interaction		Death and Dying		Intimacy	
	MX	EC	MX	EC	MX	EC
Gender						
Male	3.93 (0.88)	3.98 (0.75)	4.25 (0.87)	2.22 (1.12)	3.78 (0.75)	3.78 (0.75)
Female	3.87 (0.87)	4.04 (0.68)	4.15 (1.04)	3.32 (1.31)	3.92 (0.64)	3.92 (0.64)
*p*-value	0.687	0.635	0.493	<0.01	0.270	0.051
Age						
Between 60–69 years	3.91 (0.86)	4.16 (0.68)	4.18 (0.92)	2.86 (1.47) ^1^	3.79 (0.72)	3.75 (0.92)
Between 70–79 years	3.96 (0.95)	3.99 (0.69)	4.50 (0.77)	3.37 (1.32) ^1,2^	3.88 (0.81)	3.51 (0.96)
More than 80 years	-	4.01 (0.71)	-	2.74 (1.08) ^2^	-	3.61 (0.80)
*p*-value	0.762	0.359	<0.05	<0.05	0.465	0.284
Education						
Elementary school	-	3.85 (0.73) ^1^	-	3.14 (1.21)	-	3.47 (1.07)
High school	-	3.95 (0.77) ^2^	-	3.27 (1.34)	-	3.53 (0.99)
Undergraduate	3.93 (0.82)	4.07 (0.52) ^3^	4.15 (0.95)	3.07 (1.32)	3.75 (0.67)	3.55 (0.84)
Graduate	3.91 (0.91)	4.49 (0.57) ^1,2,3^	4.28 (0.87)	2.98 (1.61)	3.85 (0.77)	3.99 (0.65)
*p*-value	0.863	<0.01	0.254	0.719	0.318	0.195
Civil status						
Married	3.91 (0.88)	3.95 (0.76)	4.22 (0.92)	2.75 (1.30)	3.80 (0.73)	3.77 (0.80)
Single(a)	3.97 (0.84)	4.08 (0.65)	4.27 (0.78)	3.38 (1.32)	3.88 (0.76)	3.48 (0.98)
*p*-value	0.690	0.179	0.761	<0.01	0.539	<0.05
Work after retirement						
Paid job	4.09 (0.81)	4.15 (0.82)	4.14 (0.98)	2.53 (1.49)	3.89 (0.78)	3.72 (1.16)
Unpaid job	3.85 (0.90)	4.00 (0.67)	4.27 (0.86)	3.23 (1.32)	3.77 (0.71)	3.59 (0.86)
*p*-value	0.055	0.329	0.329	<0.05	0.249	0.510
Chronic disease						
Yes	3.92 (0.89)	4.07 (0.61)	4.21 (0.86)	3.12 (1.33)	3.73 (0.71)	3.54 (0.90)
No	3.92 (0.87)	3.96 (0.82)	4.24 (0.93)	3.22 (1.37)	3.86 (0.75)	3.66 (0.97)
*p*-value	0.983	0.289	0.802	0.611	0.173	0.366
Frequency of medical examinations						
More than twice a year	4.04 (0.71) ^1^	3.94 (0.68)	4.23 (0.81)	3.15 (1.21)	3.90 (0.71)	3.52 (0.85)
Once or twice a year	4.05 (0.91) ^2^	4.11 (0.64)	4.26 (0.93)	3.13 (1.44)	3.81 (0.79)	3.72 (0.96)
Less than once a year	3.72 (0.98) ^1,2^	4.09 (0.73)	4.21 (0.90)	3.20 (1.44)	3.69 (0.75)	3.55 (0.99)
*p*-value	<0.05	0.222	0.942	0.954	0.248	0.447
Sport practice						
Yes	3.99 (0.83)	4.04 (0.70)	4.20 (0.91)	3.17 (1.40)	3.88 (0.69)	3.62 (0.97)
No	3.80 (0.96)	4.02 (0.67)	4.28 (0.88)	3.12 (1.23)	3.67 (0.80)	3.50 (0.83)
*p*-value	0.120	0.851	0.516	0.801	<0.05	0.386

MX: Mexico; EC: Ecuador. (a): Includes single, divorced, and widowed older adults. Standard deviations are shown in parentheses. A *t*-test was used to compare the dimensions of quality of life among older adults grouped by gender, age (only for the Mexican sample), educational level (only for the Mexican sample), marital status, work after retirement, presence of chronic disease, and sports practice. An ANOVA test was performed to compare the scores of older adults according to age (only for the Ecuador sample), educational level (only for the Ecuador sample), and frequency of medical examinations. ^1^, ^2^, ^3^: Statistically significant differences were found between these pairs at a *p* <0.05 level.

## Data Availability

The data presented in this study are available on request from the corresponding author (P.O.P.). The data are not publicly available due to privacy concerns.
